# Preliminary IL-1 Family Cytokine Signature for Crohn’s Disease Onset in Pediatric Juvenile Idiopathic Arthritis

**DOI:** 10.3390/ijms27104247

**Published:** 2026-05-10

**Authors:** Angelina V. Polyanskaya, Anna G. Soboleva, Alexandre Mezentsev, Vladimir V. Sobolev, Svetlana N. Chebysheva, Natalia A. Geppe, Elena S. Zholobova, Maria K. Osminina, Vera A. Podzolkova, Marina D. Shakhnazarova, Olga G. Suhov’eva, Irina M. Farber, Irina M. Korsunskaya

**Affiliations:** 1 Sechenov First Moscow State Medical University (Sechenov University), 8 Trubetskaya Street, Building 2, Moscow 119048, Russia; meleshkina.angel@mail.ru (A.V.P.); svetamma@gmail.com (S.N.C.); geppe@mail.ru (N.A.G.); zholobova_1959@mail.ru (E.S.Z.); mk_osminina@mail.ru (M.K.O.); v.a.podzolkova@gmail.com (V.A.P.); marinashakh@mail.ru (M.D.S.); olga5s@mail.ru (O.G.S.); imfarber@gmail.com (I.M.F.); 2 Avtsyn Research Institute of Human Morphology, Federal State Budgetary Scientific Institution “Petrovsky National Research Centre of Surgery”, Moscow 119991, Russia; annasobo@mail.ru; 3 Center for Theoretical Problems of Physicochemical Pharmacology, Russian Academy of Sciences, Moscow 119334, Russia; vlsobolew@gmail.com (V.V.S.); marykor@bk.ru (I.M.K.); 4 Institute of Molecular and Cellular Medicine, Peoples’ Friendship University of Russia (RUDN University), Moscow 117198, Russia

**Keywords:** IL-1 family, biomarker discovery, pediatric inflammatory bowel disease, cytokine profiling, translational immunology, Crohn’s disease, juvenile idiopathic arthritis, psoriatic arthritis

## Abstract

Crohn’s disease (CD) in children with juvenile idiopathic arthritis (JIA) is frequently diagnosed late due to overlapping symptoms and non-specific biomarkers. We hypothesized that longitudinal cytokine profiling could identify a pre-symptomatic signature predictive of CD conversion in pediatric JIA patients. Ninety pediatric participants (JIA, CD, psoriatic arthritis, healthy controls) underwent serum cytokine profiling (IL-1α, IL-1β, IL-36α, IL-37, IL-6, IL-18, IL-27, IL-31) at baseline and 12 months. Statistical analysis used Mann–Whitney U tests for two-group comparisons, the Kruskal–Wallis test with Dunn’s post hoc for multi-group comparisons, Fisher’s Exact Test for categorical outcomes, and exploratory principal component analysis (PCA). Baseline screening identified a subgroup of JIA patients (*N* = 4) with significantly elevated IL-1α, IL-1β, and IL-36α. At 12 months, all four patients in this subgroup received a secondary CD diagnosis (4/4 converters vs. 0/21 non-converters; Fisher’s Exact Test: *p* < 0.0001). The longitudinal analysis at conversion revealed a broader pro-inflammatory shift, with marked increases in IL-18 and IL-31, alongside elevated IL-37, suggesting a compensatory regulatory response. PCA confirmed that converters clustered distinctly from both stable JIA and established CD. A baseline IL-1 family signature may represent a preliminary predictive signature for CD onset in pediatric JIA. Although constrained by the small converter subgroup (*N* = 4), these data support cytokine profiling for earlier diagnosis in high-risk populations.

## 1. Introduction

Crohn’s disease (CD) is an immune-mediated pathological condition of the gastrointestinal tract, initiated by a microbial agent in individuals with a genetic predisposition [1]. Most commonly presenting in young adulthood (ages 15–30), the disease follows a relapsing and remitting clinical course characterized by focal, asymmetric, and transmural inflammation that can involve any segment of the alimentary canal. These inflammatory processes frequently result in non-caseating granulomas, luminal narrowing, and perianal complications. Furthermore, its phenotypic expression is significantly shaped by environmental triggers (e.g., smoking, diet, and antibiotic exposure), which exacerbate the underlying immune dysregulation. Common clinical manifestations of CD include abdominal pain, chronic diarrhea, weight loss, and fatigue, often accompanied by fever during active disease flares, perianal disease (fissures, fistulae, abscesses), and extraintestinal manifestations such as arthralgias, uveitis, and erythema nodosum [2].

The Montreal Classification is used to stratify CD phenotypes based on age at diagnosis, anatomical location, and clinical behavior, providing a framework for prognosis and therapeutic selection. Patients are categorized by age at diagnosis into A1 (≤16 years, pediatric onset), A2 (17–40 years), and A3 (>40 years). Disease location is defined as L1 (ileal), L2 (colonic), L3 (ileocolonic), or L4 (isolated upper gastrointestinal involvement, which may coexist with L1–L3). Finally, clinical behavior is classified as B1 (non-stricturing, non-penetrating), B2 (stricturing), or B3 (penetrating), with the addition of a ‘p’ modifier to denote concomitant perianal disease [3].

The diagnosis and management of CD have evolved toward early intervention with advanced therapies and standardized classification systems to predict disease progression. Because there is no single “gold standard” test for CD, diagnosis relies on a combination of clinical, endoscopic, and radiologic evidence [4]. The most common problem is that many typical symptoms of CD such as diarrhea, abdominal pain, and weight loss are non-specific and overlap with numerous other conditions, including irritable bowel syndrome, infections, celiac disease, and ulcerative colitis. Key diagnostic challenges of CD include the inaccessibility of some affected areas (e.g., the small intestine), the patchy distribution of lesions, the difficulty in differentiating it from intestinal tuberculosis, and the limited specificity of clinically used biomarkers [5,6].

In clinical practice, diagnosing CD in patients with concurrent immune-mediated inflammatory diseases (IMIDs) such as rheumatoid arthritis, psoriasis, or ankylosing spondylitis presents unique challenges, as systemic inflammation characteristic of these conditions compromises the specificity of standard laboratory tests [7,8,9]. The primary limitation lies in the non-specific nature of routine biomarkers during simultaneous inflammatory processes. C-reactive protein (CRP), a systemic inflammation marker, may elevate due to joint or skin flares rather than intestinal activity, creating false-positive signals for CD activity [10,11]. Fecal calprotectin (FC), while more gut-specific, remains elevated in ~21% of patients with psoriatic arthritis (PsA) without inflammatory bowel disease (IBD), risking misdiagnosis in rheumatology or dermatology settings [12,13]. To bypass this “biochemical noise,” clinicians increasingly rely on cross-sectional imaging, such as magnetic resonance enterography (MRE) or intestinal ultrasound (IUS), which allows for the direct visualization of transmural bowel wall inflammation independently of systemic inflammatory markers.

The literature on pediatric populations regarding cytokine profile changes associated with the development of CD in patients with juvenile idiopathic arthritis (JIA) remains limited. Existing studies are predominantly case reports (e.g., [14]) that focus on clinical presentation and symptom management rather than investigating underlying molecular events, including the dynamic expression of cytokines. This complexity is further compounded by the dynamic nature of pediatric rheumatic phenotypes, which frequently evolve over time. For example, systemic JIA may transition from an IL-1-driven acute phase to an IL-17-driven chronic state [15].

Although shifts in cytokine profiles are increasingly recognized as potential triggers for CD in patients with underlying inflammatory disorders such as JIA, the precise molecular mechanisms driving this phenomenon remain largely elusive [16]. Pharmacological evidence supports this link: Hügle et al. [15] reported new-onset IBD following treatment with IL-1 antagonists, whereas multiple studies describe IBD emergence after the therapy with TNF-α blockers across various JIA subtypes [17,18,19]. Similarly, Fauny et al. [20] documented IBD development following anti-IL-17 therapy.

In the latter case, the authors hypothesized that the neutralization of IL-17A may compromise the integrity of intestinal epithelial barrier, thereby precipitating mucosal inflammation. Further mechanistic insights come from studies highlighting IL-24, a downstream gene of the IL-17 signaling pathway [21], which acts as a regulator of mucosal inflammation by modulating the expression of membrane-bound mucins (MUC1, MUC3, and MUC4) [22]. Because these critical cytokine shifts remain poorly characterized, cytokine-targeted biologics should be used with caution in patients predisposed to IBD. Conversely, monitoring baseline cytokine profiles associated with subclinical CD may provide a valuable tool for early risk stratification prior to or during the initial phases of biologic therapy.

Due to early onset of CD, pediatricians and rheumatologists maintain a high suspicion for children with persistent joint pain unresponsive to standard arthritis therapies, even without classic gastrointestinal symptoms. Recently, Yablokova et al. [23] highlighted musculoskeletal disorders as frequent extraintestinal manifestations of pediatric CD and ulcerative colitis, driven by the gut–joint axis and increased intestinal permeability. A recent study of 146 pediatric patients [7] found that joint syndrome is a highly common extraintestinal manifestation in pediatric IBD, with half of children exhibiting both joint and bowel symptoms experiencing joint pain prior to gastrointestinal onset, a pattern particularly prevalent in CD, often leading to years of misdiagnosis as juvenile arthritis.

In this study, we report the novel observation that JIA patients exhibit elevated serum levels of pro-inflammatory cytokines (IL-1α, IL-1β, and IL-36α) indicative of subclinical CD. These cytokine signatures distinguish JIA to CD converters from only JIA- patients and the healthy control (HC), potentially enabling earlier CD diagnosis in at-risk rheumatology cohorts. Moreover, this cytokine axis represents promising dual therapeutic targets for managing co-existing JIA and CD.

## 2. Results

**Figure 1:**
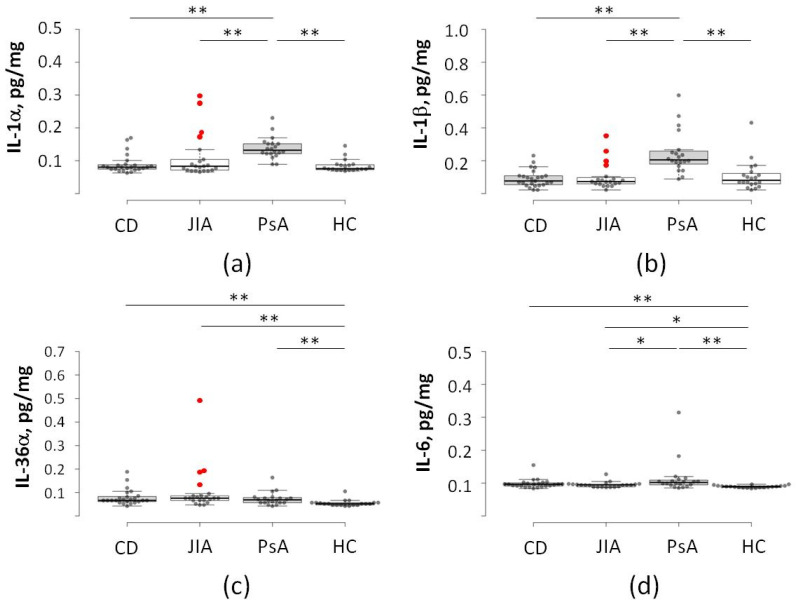
Baseline predictive cytokine signature in JIA patients. (**a**) IL-1α, (**b**) IL-1β, (**c**) IL-36α, and (**d**) IL-6. Baseline serum cytokine levels across the four study cohorts (CD, JIA, PsA, and HC). Note the significant elevation of IL-1 family members in a specific JIA subgroup (*N* = 4, marked in red), providing a distinct pro-inflammatory profile prior to the clinical onset of secondary disease. CD—Crohn’s disease (*N* = 25); JIA—juvenile idiopathic arthritis (*N* = 25; non-converters, *N* = 21); PsA—psoriatic arthritis (*N* = 20); HC—healthy control (*N* = 20). *—*p* < 0.05; **—*p* < 0.01.

Despite well-documented associations between specific cytokine abnormalities and distinct autoimmune disorders, an elevated pro-inflammatory background can play a dual role: it may either mask subclinical diagnoses that emerge later or serve as a marker for increased disease severity. Although exploratory cytokine screening may occasionally reveal such abnormalities, the clinical consequences are heavily dependent on patient-specific variables, including genetic predisposition and environmental trigger factors. As presented in Figure 1, the patient cohorts exhibited significant differences in the expression of specific cytokines. The disease groups CD, JIA, and PsA showed significantly higher levels of IL-6 and IL-36α compared to HC. Notably, patients with PsA demonstrated a generalized elevation across all tested cytokines. Cohort heterogeneity remains a critical factor influencing statistical outcomes. For instance, the levels of IL-36α would not differ significantly between JIA and PsA groups if the higher expression of IL-36α observed in a small sub-cohort of JIA patients were excluded. Conversely, the marked abnormalities in the expression of IL-1α and IL-1β within this specific subgroup stand in stark contrast to the significantly lower levels of these cytokines observed in the remainder of the JIA cohort.

**Figure 2:**
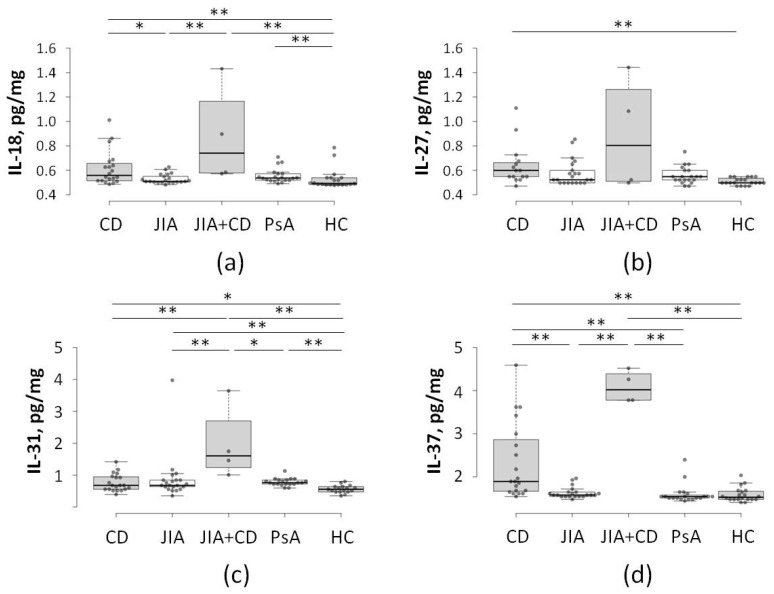
Cytokine profile shift at 12-month follow-up (Crohn’s conversion). (**a**) IL-18, (**b**) IL-27, (**c**) IL-31, and (**d**) IL-37. Comparative analysis of cytokine levels in JIA converters at the time of Crohn’s disease diagnosis. Data demonstrate a broad expansion of the inflammatory cascade (IL-18, IL-31) alongside a compensatory increase in the anti-inflammatory mediator IL-37, reflecting active systemic inflammation and immune regulation. CD—Crohn’s disease (*N* = 25); JIA—juvenile idiopathic arthritis (*N* = 25); JIA + CD—JIA patients diagnosed with CD (*N* = 4); PsA—psoriatic arthritis (*N* = 20); HC—healthy control (*N* = 20); *—*p* < 0.05; **—*p* < 0.01.

A follow-up study conducted on the same patient population twelve months later revealed further abnormalities in cytokine profiles (Figure 2). During this period, the JIA sub-cohort (*N* = 4) was re-classified as “CD + JIA” because these patients had received a clinical diagnosis of CD. In this second phase of analysis, which focused on a different panel of pro-inflammatory mediators, this transitioning group was distinguished from other cohorts by a significantly higher expression of IL-31 (Figure 2). This subgroup also exhibited elevated levels of IL-37 compared to both standard JIA and PsA patients, aligning more closely with the expression range typical of established CD patients. In contrast, no significant differences were detected between the new “CD + JIA” cohort and the other groups regarding the expression of IL-27.

To validate the predictive power of the cytokine signature identified in the initial JIA cohort, Fisher’s Exact Test was performed. The results demonstrated perfect separation: all four patients with elevated baseline IL-1α, IL-1β, and IL-36α levels converted to CD (4/4, 100%), whereas none of the remaining 21 JIA patients did (0/21, 0%; Fisher’s Exact Test: *p* < 0.0001). These findings suggest complete separation of the cohorts in this pilot sample when predicting CD conversion.

**Figure 3:**
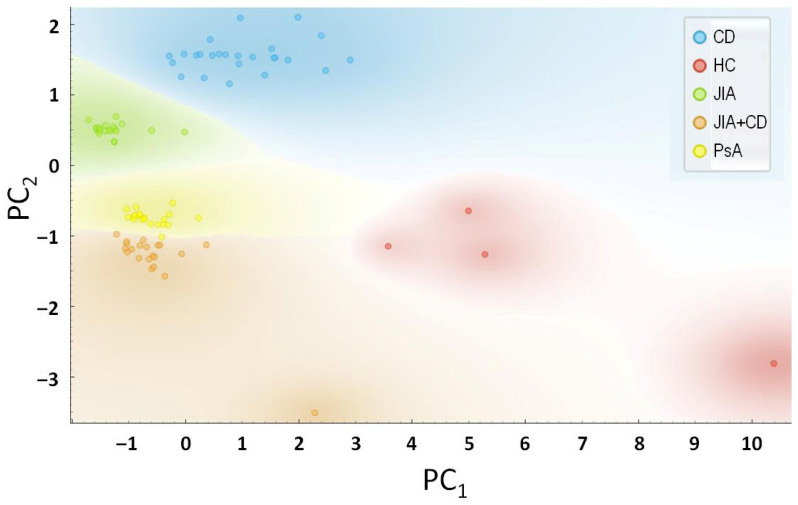
Principal component analysis at 12-month follow-up (Crohn’s conversion) in 90 pediatric patients based on a selected cytokine panel. The JIA patients diagnosed with CD (*N* = 4) cluster distinctly from the standard JIA cohort, showing a signature closely aligned with neither group. This figure shows complete separation of the “high-cytokine” subgroup over the 12-month study period. CD: Crohn’s disease (*N* = 25); JIA: juvenile idiopathic arthritis (*N* = 21); CD + JIA: JIA patients diagnosed with CD (*N* = 4); PsA: psoriatic arthritis (*N* = 20); HC: healthy control (*N* = 20).

The results of principal component analysis (PCA) presented in Figure 3 further confirmed the distinct biological identity of this high-risk subgroup. Principal component 1/PC1 (explaining 34.4% of the variance) and PC2 (16.3% of the variance) clearly clustered the “converters” separately from both non-converters and established CD patients. This transitional cytokine signature, which is neither purely JIA nor CD, identifies IL-1 family cytokines as robust early-warning biomarkers. Such signatures may allow for the identification of subclinical CD in at-risk JIA populations, though these results warrant further validation in larger, multi-center prospective cohorts.

## 3. Discussion

In this study, we conducted a longitudinal analysis of the cytokine profiles associated with the clinical progression of CD in a predisposed cohort of JIA patients. By screening 90 pediatric patients, we identified a baseline “pre-conversion” signature characterized by significant elevations in IL-1α, IL-1β, and IL-36α (Figure 1). Remarkably, this specific inflammatory background showed complete predictive performance for future CD diagnosis within our JIA cohort. A 12-month follow-up investigation revealed a secondary cytokine shift upon clinical conversion, marked by the emergence of IL-31 and IL-18, alongside a compensatory increase in the anti-inflammatory mediator IL-37 (Figure 2). These results suggest a phased immune evolution, transitioning from a systemic IL-1-driven state to a more complex, tissue-specific inflammatory landscape as the secondary disease manifests. We emphasize that these interpretations derive from serum cytokine measurements alone and do not constitute direct mechanistic evidence. Rather, they align with prior studies linking IL-1/IL-31 axis dysregulation to barrier dysfunction, generating testable hypotheses for future tissue-level investigations.

At the molecular level, the transition from systemic joint inflammation to localized intestinal pathology represents a breakdown in innate immune homeostasis, permitting systemic inflammatory signals to manifest as localized gut pathology [14]. The IL-1 superfamily of 11 cytokines is crucial for both host defense and autoinflammatory progression. Pro-inflammatory members, such as IL-1β and IL-18, require processing by the NLRP3 inflammasome, a multi-protein complex that senses cellular stress and microbial molecular patterns. In the context of JIA, a persistent activation of inflammasomes may not only drive synovial inflammation but may also be associated with increased mucosal susceptibility, potentially through pathways involving epithelial barrier proteins or altering the local myeloid cell rheostat, facilitating the transition to CD [24]. Emerging evidence suggests that IL-36α, a more recently characterized member of the IL-1 family, acts as a potent amplifier of this response by inducing the expression of various chemokines and metalloproteinases in both joints and gut [25].

In our cohort, the baseline elevation of IL-1α, IL-1β, and IL-36α preceded clinical CD diagnosis by 12 months, suggesting that dysregulation of this inflammasome-linked axis may represent an early immunological turning point, not solely a correlative marker. This aligns with evidence that IL-36α amplifies the release of chemokines in colonic myofibroblasts [25,26], and extends it by positioning them as potential upstream nodes in a shared JIA–CD autoinflammatory cascade. Furthermore, the balance between pro- and anti-inflammatory signaling is maintained by regulatory cytokines like IL-37. IL-37 is unique within the IL-1 family as it translocates to the nucleus to inhibit the transcription of pro-inflammatory genes, serving as a “natural brake” on systemic inflammation. However, in chronic IMIDs, the IL-37-mediated regulatory axis often becomes functionally overdrawn or maladapted; despite compensatory elevations in IL-37, the anti-inflammatory signaling fails to neutralize the escalating pro-inflammatory burden, leading to sustained tissue pathology [27,28].

Another pro-inflammatory cytokine analyzed in this study, IL-31, is traditionally associated with pruritus [29]. However, it may recruit neutrophils via induction of CXCL8 in epithelial cells [30]. For this reason, its elevation in JIA patients suggests its potential role in the systemic-to-local inflammatory transition observed in subclinical CD. Understanding the temporal shift from IL-1-driven “pre-clinical” states to an IL-31/IL-37-dominant active state can be essential for developing the next generation of preliminary predictive signatures.

These molecular findings must be contextualized within current clinical diagnostic frameworks. The following distinct clinical and laboratory markers differentiate JIA from joint syndrome of CD in pediatric patients [6]. JIA more frequently affects girls. In many cases, it involves the knees, ankles, and wrists. In turn, CD-associated joint syndrome more often affects boys. It presents with asymmetric lower limb involvement and enthesitis, and is associated with lower rates of positive antinuclear factor (25% vs. 69.6% in JIA). FC testing and sacroiliitis further aid distinction. Additionally, joint syndrome in children with both IBD and PsA differs from isolated PsA [8]. In this combined group, CD predominates, with asymmetric oligoarthritis common at onset (75%) and peak (50%), whereas isolated PsA typically progresses from oligo- to polyarthritis affecting the knees and ankles. However, since traditional adult diagnostic criteria often fall short in children, necessitating pediatric-specific tools like the Garmisch–Partenkirchen criteria [31], there is a need for a new set of preventive biomarkers that, relying on non-invasive techniques, could identify pediatric JIA patients suspicious for underlying intestinal syndromes.

The identification of elevated IL-1 family members as a predictive signature suggests that the transition to CD may be preceded by a specific dysregulation of the innate immune system that precedes clinical gastrointestinal symptoms [32]. Although IL-1β is a well-known driver of systemic JIA, its role as a precursor to CD suggests a shared autoinflammatory origin. IL-1 family members are unique because they act similar to “alarmins”—molecules released during cellular stress that trigger a massive innate immune response [33]. In our cohort, all individuals converted, suggesting that these cytokines may represent upstream nodes in a shared autoinflammatory cascade rather than general markers of inflammation. This aligns with evidence [34] highlighting the IL-1 family as key mediators of disrupting the intestinal epithelial barrier. Specifically, IL-36α has been shown to induce pro-inflammatory cytokines in colonic myofibroblasts [26], suggesting that “high-risk” JIA patients may experience subclinical mucosal stress long before clinical onset. Consequently, these findings support the hypothesis that a JIA + CD subgroup shares a common autoinflammatory origin, where the IL-1 pathway serves as the primary immunological transition point for systemic progression.

The specific elevation of IL-36α (Figure 1) is particularly noteworthy. Unlike IL-1β, which is ubiquitous, IL-36α is highly expressed in epithelial barriers, specifically the skin and the intestinal mucosa. In our cohort, the elevated baseline levels of IL-36α in the “converters” group likely reflect subclinical mucosal inflammation that was occurring months before the appearance of diarrhea or weight loss. The studies by Medina-Contreras et al. [35] have shown that IL-36 signaling boosts the recruitment of neutrophils to the gut and impairs the healing of the intestinal lining. Therefore, we propose that JIA patients with this specific “IL-1 high” profile represent a distinct clinical phenotype, one which may involve heightened NLRP3 inflammasome activity that precedes clinical gastrointestinal involvement from the synovium to the alimentary canal.

As the disease progresses, the accumulation of systemic pro-inflammatory cytokines (IL-1, IFN-γ, TNF-α), alongside microbial antigens like lipopolysaccharides (LPSs), likely triggers the upregulation of IL-31. In this context, IL-31 acts as a mechanistic bridge between active immune response and structural remodeling. By targeting intestinal epithelial cells (IECs) and subepithelial myofibroblasts (SEMFs), IL-31 promotes a dual state of inflammation and fibrosis [36]. Previous studies demonstrate that IL-31 coordinates responses across the epithelium and stroma by activating matrix metalloproteinases (MMP-1, -3, -7, and -25), which degrade the extracellular matrix, and inducing chemokines such as CXCL8 and CCL15 [30]. This disruption of barrier function contributes to the “leaky gut” phenotype characteristic of IBD, whereas its synergistic effect with IL-17A may further drive the development of CD strictures. The selective elevation of IL-31 in our “CD + JIA” subgroup (Figure 2), but not in the stable JIA cohort, highlights it as a potential “conversion marker.” This finding provides a biological explanation for why traditional biomarkers like CRP fail. Although CRP reflects general systemic stress, IL-31 specifically marks the pathological transition toward a tissue-destructive, mucosa-focused inflammatory profile.

Critically, we observed a marked induction of IL-18, likely driven by genetic predisposition and microbial triggers (Figure 2). In the transitioning CD environment, infiltrating macrophages in the inflamed mucosa increase NF-κB-mediated transcription of *IL18* and caspase-1 processing of pro-IL-18 [37]. Once secreted, IL-18 synergizes with IL-12 to drive Th_1_ response, culminating in compromised epithelial integrity, bacterial translocation, and ulceration [38]. The co-induction of IL-18 and IL-31 thus creates a suitable environment that makes genetically predisposed JIA patients highly vulnerable to CD initiation.

In addition, our follow-up experiments revealed concurrent IL-37 elevation during CD conversion (Figure 2). As a potent anti-inflammatory IL-1 family member, IL-37 functions as a “natural brake,” suppressing the production of IL-6 and TNF-α by blocking IL-18 signaling via the IL-18Rα/IL-1R8 complex. This coincidental rise likely represents late-stage compensatory negative feedback triggered by pro-inflammatory cytokines. However, despite this response, progression persisted in our cohort. Intriguingly, our “converters” exhibited persistently elevated serum IL-37, contrasting with the reduced levels characteristic of established CD (Figure 2). This “IL-37-high” phenotype may represent a unique feature of pediatric inflammatory transition, positioning IL-37 as a critical biomarker for identifying at-risk JIA patients during the clinical conversion window.

In healthy individuals, IL-37 is induced in response to inflammatory signals and translocates to the nucleus, where it suppresses the transcription of pro-inflammatory genes [39]. However, during emerging CD flares, the overwhelming cytokine storm (involving IL-18 and, in our model, IL-31) likely outpaces the regulatory capacity of IL-37. This “failed compensation”, a hallmark of chronic IMIDs, explains the need for advanced biologics to achieve mucosal healing. Our PCA (Figure 3), in turn, confirms this finding, with converters clustering in a distinct space defined by simultaneous elevation of both inflammatory drivers and exhausted regulatory responses.

Based on the perfect separation demonstrated by Fisher’s Exact Test and PCA, we propose that this exploratory biomarker framework could inform future screening strategies for JIA. Currently, pediatric rheumatologists monitor for CD based on symptoms (weight loss, abdominal pain). We suggest that routine cytokine screening for the proposed “IL-1/IL-36 signature” could identify high-risk “converters” up to a year in advance. This would allow for: early referral to gastroenterology for MRE, preemptive use of exclusive enteral nutrition (EEN), and selection of “dual-action” biologics (such as anti-IL-1 or anti-TNF-α agents) that treat both joint and gut pathology simultaneously.

The present study possesses several distinct strengths. By tracking the same pediatric patients from a baseline JIA diagnosis through to the clinical onset of CD, we were able to capture unexpected changes in the cytokine landscape, a perspective often missed in cross-sectional studies. Furthermore, the inclusion of disease-control groups (PsA and established CD) allowed us to filter out general inflammatory markers and isolate a signature specific to the JIA-to-CD conversion process.

The proposed IL-1/IL-36 signature could inform risk-stratified treatment selection in pediatric JIA patients at risk for CD conversion. For example, early identification of high-risk patients might support preemptive referral for exclusive enteral nutrition (EEN) or consideration of ‘dual-action’ biologics (e.g., anti-IL-1 or anti-TNF-α agents) that target both synovial and intestinal inflammation [23,38,39]. This biomarker-guided approach aligns with current treat-to-target strategies while potentially enabling earlier intervention before irreversible mucosal damage occurs.

At the same time, several limitations must be acknowledged. First, the size of our “converter” subgroup (*N* = 4) is small. Although the statistical significance achieved via Fisher’s Exact Test (*p* < 10^−4^) and the complete separation in PCA are robust, these results represent a pilot cohort. The complete cohort separation observed here may reflect overfitting and should be validated in larger, multicenter prospective trials to establish universal clinical thresholds. The cytokine pattern was identified retrospectively after clinical conversion was confirmed. Because no pre-defined cut-offs were established and the analysis was performed post hoc, the observed separation represents a hypothesis-generating signal rather than a validated diagnostic threshold. Prospective studies with pre-specified thresholds and independent cohorts are required before clinical consideration can be justified.

Second, the current study focused on serum concentrations of cytokines. Although serum provides a non-invasive window into systemic inflammation, it does not always accurately reflect the localized microenvironment of the intestinal mucosa [40,41,42] or synovial fluid [43]. Future studies would provide a deeper mechanistic understanding of the IL-1/IL-31 axis at the tissue level. Baseline IL-18 levels were not assessed in this pilot cohort. Consequently, it remains unclear whether IL-18 elevation preceded clinical conversion or emerged during disease progression. Prospective studies utilizing broader baseline cytokine panels are needed to clarify the temporal relationship between IL-18 dynamics and CD onset in JIA patients.

Third, medication history of the patients, including the use of non-steroidal anti-inflammatory drugs (NSAIDs) or methotrexate, may influence baseline cytokine levels [44,45]. Although our cohorts were age-matched, the heterogeneity of JIA phenotypes (e.g., oligoarticular vs. polyarticular) remains a potential confounding factor. Despite these limitations, the strength of the observed predictive signal provides a compelling rationale for further investigation into the “pre-clinical” phase of pediatric CD.

We also acknowledge the gender imbalance within our JIA cohort (81% female). Although a female bias is epidemiologically typical for many JIA subtypes [46,47], early-onset CD often presents with a male preponderance in pediatric populations [48]. Due to immune signatures, including neutrophil maturation and cytokine pathways can exhibit sexual dimorphism [49,50], the high prevalence of females in our study may limit the generalizability of the ‘pre-conversion’ signature to male patients. Future large-scale studies should aim for a more balanced gender distribution to determine if these exploratory biomarker candidates remain robust across sexes.

As a pilot biomarker discovery study, this work did not include internal cross-validation or external validation. Future studies will use resampling approaches and multicenter cohorts to evaluate the robustness and generalizability of the findings.

## 4. Materials and Methods

### 4.1. Ethics Statement

All biological samples were collected from pediatric patients and healthy volunteers after obtaining informed written consent from their parents or legal guardians. The experimental procedures were conducted in strict accordance with the ethical principles of the Declaration of Helsinki and complied with all relevant national regulations regarding human subject research. The study protocol was reviewed and formally approved by the Ethics Committee at the Center of Theoretical Problems of Physicochemical Pharmacology (Approval Date: 12 February 2024; Protocol #2).

### 4.2. Study Cohort

A total of 90 participants were enrolled in this study, comprising four groups: patients with CD (*N* = 25), patients with JIA (*N* = 25), patients with PsA (*N* = 20), and HC (*N* = 20). Notably, the JIA cohort included four individuals who subsequently received a secondary diagnosis of comorbid CD during the 12-month follow-up period. Accordingly, the converter subgroup (*N* = 4) was identified retrospectively: all JIA patients underwent baseline serum collection prior to a 12-month clinical follow-up, during which CD diagnosis was established independently using standardized endoscopic and histological criteria. Their baseline cytokine profiles (IL-1α, IL-1β, and IL-36α) were descriptively characterized as “elevated” relative to the JIA non-converter median and healthy control range; no pre-specified numerical thresholds were applied. This retrospective, hypothesis-generating approach is consistent with the pilot design of this study. Consequently, these findings should be interpreted as exploratory signals requiring validation in pre-specified, multicenter prospective cohorts.

The diagnosis of CD in patients with JIA was established according to the standard diagnostic algorithm approved by the Scientific and Practical Council of the Russian Ministry of Public Health in 2024 [51]. Briefly, the diagnostic workup included ileocolonoscopy with biopsy of the terminal ileum, followed by esophagogastroduodenoscopy. The endoscopic and histological findings met the diagnostic criteria for Crohn’s disease with ileitis and colitis (L3) including documentation of non-caseating granulomas and/or transmural inflammation where present, according to the Paris classification (2010) [52]. JIA subtypes were classified according to clinical practice at our center, distinguishing oligoarticular (3–4 joints) and pauciarticular (1–2 joints) presentations, as defined in the Union of Pediatricians of Russia clinical guidelines [53]. All classifications were confirmed by board-certified pediatric rheumatologists.

Children aged 6–16 years with confirmed diagnoses were eligible: JIA (including psoriatic JIA) per ILAR criteria [54]; CD per Paris pediatric IBD classification [52]; and PsA (non-JIA) per CASPAR criteria [55]. Inclusion required active disease requiring systemic therapy, no prior concurrent autoimmune/autoinflammatory conditions (beyond index diagnosis), availability of a baseline serum sample, and 12-month follow-up commitment. Healthy controls were age- and sex-matched individuals without chronic inflammatory conditions. Exclusion criteria were the following: concurrent conditions beyond the index diagnosis, active infection/malignancy, recent immunosuppression, or incomplete data. Consecutive enrollment occurred at Filatov Clinical Institute of Children’s Health, Sechenov University, between 13 February 2024 and 12 March 2024.

Comprehensive demographic and clinical data for all participants, including gender, age at enrollment, age at primary diagnosis, and duration of clinical observation, are detailed in Supplementary Table S1. Briefly, there was no significant difference between the cohorts on age and duration of clinical observation. The groups were gender-balanced, except patients with JIA where females outnumbered males (4:1), reflecting known female predominance [46,47].

### 4.3. Blood Samples

Venous blood samples were collected from all participants (*N* = 90) between 8:00 AM and 10:00 AM to minimize circadian variation in cytokine expression. Samples were collected in sterile, EDTA-free vacutainer tubes (MiniMed, Suponevo, Russia) and allowed to clot at room temperature for 30 min. Following centrifugation at 1000–2000× *g*, 4 °C, 10 min, serum was aliquoted into cryovials and immediately stored at −80 °C until biochemical analysis. No samples underwent more than one freeze–thaw cycle prior to measurement. Total protein concentration in each serum sample was determined using a standard protein assay to allow for the normalization of cytokine levels per milligram of total protein, ensuring that variations in hydration status or systemic protein levels did not confound the results.

### 4.4. ELISA

Cytokine concentrations were determined via sandwich ELISA using commercial kits for IL-1A (#SEA071Hu), IL-1B, (#SEA563Hu), IL-36α/IL-1E (#SEE843Hu), IL-37/IL-1Z (#SEE842Hu), IL-6 (#SEA079Hu), IL-18 (#SEA064Hu), IL-27A (#SEA385Hu), and IL-31 (#SEB179Hu) supplied by Cloud-Clone Corp. (Katy, TX, USA). All reagents and samples were brought to room temperature before use. Briefly, 100 μL of standard or sample (appropriately pre-diluted in the provided Assay Diluent) was added to the pre-coated microplate wells, covered with a plate sealer, and incubated for 1 h at 37 °C. Following liquid aspiration (without washing), 100 μL of Detection Reagent A working solution was added and incubated for an additional hour at 37 °C. The wells were then washed three times with 350 μL of Wash Solution using an automated plate washer (or multichannel pipette), with a 1–2 min soak time between washes. The remaining wash buffer was removed by tapping the plate onto absorbent paper. Next, 100 μL of Detection Reagent B working solution was added, followed by a 30 min incubation at 37 °C. After five subsequent wash cycles, 90 μL of Substrate Solution was added to each well and incubated for 10–20 min at 37 °C until the desired color intensity developed. The reaction was quenched with 50 μL of Stop Solution, turning the solution yellow. Optical density was measured immediately at 450 nm using a Bio-Rad iMark Microplate Reader (Catalog #168-1135; Hercules, CA, USA). Analyte concentrations were calculated using a standard calibration curve. The results were normalized to the level of total protein and are reported as pg/mg total protein to account for inter-individual variation in serum protein content [56]. Baseline cytokine profiling was limited to the IL-1 family axis (IL-1α, IL-1β, and IL-36α) and IL-6 based on prior mechanistic hypotheses. IL-18, IL-27, IL-31, and IL-37 were assessed at the 12-month follow-up to explore longitudinal dynamics but were not included in the baseline discovery panel, due to sample volume constraints and the focused exploratory design of this pilot phase.

### 4.5. Protein Assay

Total protein concentrations were determined using the Quick Start Bradford Protein Assay Kit (Catalog #500-0201; Bio-Rad) with bovine serum albumin (BSA) as the standard. Reaction mixtures were prepared by combining 20 µL of protein samples (properly diluted in lysis buffer) with 1× dye reagent (1 mL) in disposable 1.5 mL microcentrifuge tubes. The reagent was allowed to equilibrate to room temperature and inverted five times before use. After a 5 min incubation at room temperature, the samples were briefly vortexed and transferred to 1.5 mL disposable cuvettes, and the optical density was measured at 595 nm using a Hitachi U-557 spectrophotometer (Hitachi, Japan), blanked against a 1× dye reagent/buffer mixture. Protein concentrations were calculated from a 7-point standard calibration curve (0.125–2.0 mg/mL BSA) generated in duplicate. All samples were assayed in triplicate.

### 4.6. Statistics

Descriptive statistics were calculated for all cohorts, with data expressed as mean ± standard error of the mean (SEM) or median with interquartile range (IQR) where appropriate. Due to the small size of the JIA converter subgroup (*N* = 4) and the non-normal distribution of cytokine concentrations, non-parametric analysis was prioritized. Baseline differences in cytokine levels across the four primary cohorts (JIA, CD, PsA, and HC) were evaluated using the Kruskal–Wallis test, followed by Dunn’s post hoc test with Šidák correction for multiple comparisons. Due to the small size of the JIA converter subgroup (*N* = 4), its baseline cytokine profile was analyzed descriptively to explore patterns associated with subsequent CD conversion. As an exploratory analysis, Fisher’s Exact Test was employed to examine the association between baseline cytokine patterns and 12-month conversion to CD. Principal component analysis (PCA) was used to visualize cohort separation; data were log-transformed and auto-scaled prior to PCA to ensure that high-abundance cytokines did not disproportionately influence the model. All *p*-values were two-tailed, and values of *p* < 0.05 were considered statistically significant. All statistical analyses were performed using Orange Data Mining 3.40 software [57].

## 5. Conclusions

In conclusion, our study identifies a preliminary biomarker signal warranting further validation for the development of CD in pediatric JIA patients. We demonstrate that the transition from joint-centered inflammation to systemic gastrointestinal disease is not a sudden event, but rather a phased immunological progression. The elevation of IL-1α, IL-1β, and IL-36α serves as a potent predictive signature, identifying “converters” up to 12 months before the manifestation of clinical symptoms. Our findings indicate that baseline elevations in IL-1α, IL-1β, and IL-36α were uniquely associated with patients who later developed clinical CD, achieving complete cohort separation in this pilot sample.

Our data further highlights a significant cytokine “shift” during disease conversion, characterized by the emergence of IL-31 and IL-18, markers likely associated with mucosal barrier breakdown and tissue remodeling. The concurrent rise in IL-37 suggests a localized but failing homeostatic response, pointing toward a window of opportunity where therapeutic intervention, perhaps through dual-action biologics, could potentially arrest the progression of the secondary disease. In addition, the ability to identify “converters” through non-invasive serum profiling before the onset of gastrointestinal symptoms provides a critical window for personalized therapeutic strategies. Early screening in JIA populations could significantly improve long-term outcomes by enabling more vigilant monitoring or preemptive adjustments in treatment. In this context, the distinct “transitional” profile identified via PCA confirms that patients diagnosed with JIA and CD represent a unique biological subgroup rather than a simple overlap of two existing conditions, highlighting the role of the IL-1 family in driving this specific disease trajectory.

Ultimately, these findings move us closer to a precision medicine approach in pediatric rheumatology. By integrating routine cytokine profiling into the monitoring of high-risk JIA populations, clinicians can bypass the ‘biochemical noise’ of standard inflammatory markers like CRP. This shift from “reactive” to “preemptive” diagnosis holds the potential to significantly reduce the morbidity associated with delayed Crohn’s diagnosis, ensuring better growth outcomes and long-term quality of life for these complex pediatric patients.

## Data Availability

The original contributions presented in this study are included in the article/Supplementary Materials. Further inquiries can be directed at the corresponding author.

## Supplementary Materials

The following supporting information can be downloaded at https://www.mdpi.com/article/10.3390/ijms27104247/s1.

## Abbreviations

The following abbreviations are used in this manuscript:

CD	Crohn’s disease
CRP	C-reactive protein
EEN	exclusive enteral nutrition
FC	fecal calprotectin
HC	healthy control
IBD	inflammatory bowel disease
IEC	intestinal epithelial cell
IMID	immune-mediated inflammatory disease
IQR	interquartile range
IUS	intestinal ultrasound
JIA	juvenile idiopathic arthritis
MMP	matrix metalloproteinase
MRE	magnetic resonance enterography
NSAID	non-steroidal anti-inflammatory drugs
PC	principal component
PCA	principal component analysis
PsA	psoriatic arthritis
SEM	standard error of the mean
SEMF	subepithelial myofibroblast
T2T	treat-to-target
TDM	therapeutic drug monitoring
