# Supplementary material for: Preliminary IL-1 Family Cytokine Signature for Crohn’s Disease Onset in Pediatric Juvenile Idiopathic Arthritis
Source: Int J Mol Sci. 2026 May 10;27(10):4247. doi: 10.3390/ijms27104247 (PMC13208062; doi:10.3390/ijms27104247)
Supplement: Supplementary file 1 [file ijms-27-04247-s001.zip › ijms-4279490-supplementary.pdf]

Table S1. Demographic data of individual participated in the study

#	Sex[1]	Diagnosis	JIA Subtype	Age[2]	Observation[3]	#	Sex[1]	Diagnosis	JIA Subtype	Age[2]	Observation[3]
1	F	CD		11	1	21	F	JIA	PA	12	6
2	F	CD		13	4	22	M	JIA+CD	OA	14	9
3	F	CD		14	7	23	F	JIA+CD	OA	11	5
4	M	CD		10	1	24	F	JIA+CD	OA	13	8
5	M	CD		16	6	25	M	JIA+CD	OA	14	8
6	F	CD		9	0	1	M	PsA		8	4
7	M	CD		11	4	2	F	PsA		12	8
8	M	CD		13	3	3	M	PsA		13	0
9	M	CD		15	6	4	F	PsA		11	7
10	F	CD		14	7	5	F	PsA		10	6
11	F	CD		10	2	6	M	PsA		11	7
12	M	CD		11	4	7	F	PsA		13	0
13	M	CD		12	3	8	M	PsA		14	1
14	F	CD		13	0	9	F	PsA		11	7
15	M	CD		9	3	10	M	PsA		12	8
16	F	CD		8	0	11	F	PsA		13	0
17	M	CD		15	7	12	M	PsA		14	9
18	F	CD		13	6	13	F	PsA		11	6
19	F	CD		12	0	14	M	PsA		10	4
20	M	CD		10	3	15	F	PsA		15	2
21	M	CD		13	0	16	F	PsA		13	9
22	M	CD		13	5	17	F	PsA		12	8
23	F	CD		10	3	18	F	PsA		17	4
24	M	CD		11	6	19	M	PsA		11	7
25	F	CD		10	5	20	M	PsA		12	8
1	F	JIA	PA	12	6	1	F	HC		9	N/A
2	F	JIA	PA	13	8	2	M	HC		10	N/A
3	M	JIA	PU	7	2	3	M	HC		11	N/A
4	M	JIA	OA	8	4	4	F	HC		9	N/A
5	M	JIA	PA	12	6	5	M	HC		8	N/A
6	F	JIA	PA	14	9	6	M	HC		10	N/A
7	F	JIA	PA	13	6	7	M	HC		10	N/A
8	F	JIA	PU	8	3	8	M	HC		12	N/A
9	M	JIA	PA	10	5	9	M	HC		9	N/A
10	F	JIA	PA	12	6	10	M	HC		10	N/A
11	F	JIA	PA	13	9	11	F	HC		11	N/A
12	F	JIA	PU	8	0	12	F	HC		12	N/A
13	F	JIA	PU	7	2	13	M	HC		8	N/A
14	F	JIA	PA	15	10	14	F	HC		7	N/A
15	F	JIA	PA	14	8	15	F	HC		13	N/A

16	F	JIA	PA	12	7	16	M	HC	11	N/A
17	F	JIA	PA	12	8	17	M	HC	10	N/A
18	F	JIA	PA	13	6	18	M	HC	11	N/A
19	F	JIA	PA	14	9	19	M	HC	9	N/A
20	F	JIA	PA	9	5	20	F	HC	10	N/A

[1] Sex at birth (M – male; F – female); [2] Age at the beginning of the first study; [3] Duration of clinical observation; CD – Crohn's disease; JIA – juvenile idiopathic arthritis; JIA+CD – JIA patients diagnosed with CD; PsA – psoriatic arthritis; HC – healthy control; N/A – not applicable. Subtypes of JIA: PA – polyarticular; OA - oligoarticular; PU – pauciarticular;
